# Synthesis and characterization of novel histidine functionalized chitosan nanoformulations and its bioactivity in tomato plant

**DOI:** 10.1038/s41598-024-64268-1

**Published:** 2024-07-02

**Authors:** Mahendra Meena, Vinod Saharan, K. K. Meena, Balraj Singh, Shalini Pilania, N. K. Gupta, Ajay Pal, O. P. Garhwal, Y. K. Sharma, Uadal Singh, Rajesh Bagri, M. K. Sharma, Rachna Sharma, B. L. Jakhar, Piyush Chandel, Damyanti Prajapati, Kinjal Mondal, Mital Mahala, D. K. Bairwa, Madhu Bai Meena

**Affiliations:** 1 Department of Horticulture, SKNCOA, SKNAU, Jobner, Rajasthan 303 329 India; 2 Department of Molecular Biology and Biotechnology, Rajasthan College of Agriculture, MPUAT, Udaipur, Rajasthan India; 3 Department of Horticulture, Rajasthan Agricultural Research Institute, SKNAU, Jobner, Rajasthan India; 4 Department of Horticulture, Rajasthan College of Agriculture, MPUAT, Udaipur, Rajasthan India; 5 Department of Plant Physiology, SKNAU, Jobner, Rajasthan India; 6 Department of Biochemistry, College of Basic Sciences and Humanities, Chaudhary Charan Singh Haryana Agricultural University, Hisar, Haryana 125 004 India; 7 Department of Plant Pathology, Rajasthan Agricultural Research Institute, SKNAU, Jobner, Rajasthan India; 8 Department of Chemistry, Dr B R Ambedkar NIT, Jalandhar, 144 011 India; 9 Department of Entomology, Rajasthan Agricultural Research Institute, SKNAU, Jobner, Rajasthan India; 10 Department of Entomology, SKNCOA, SKNAU, Jobner, Rajasthan 303 329 India; 11 Department of Plant Pathology, Rajasthan College of Agriculture, MPUAT, Udaipur, Rajasthan India

**Keywords:** Chitosan–histidine nanoformulations, Chitosan, Histidine, Cross-linked, Tomato, Biochemistry, Biotechnology, Chemical biology, Molecular biology, Physiology, Plant sciences, Structural biology, Chemistry, Materials science, Nanoscience and technology

## Abstract

The use of novel active ingredients for the functional modification of chitosan nanoformulations has attracted global attention. In this study, chitosan has been functionalized via histidine to craft novel chitosan–histidine nanoformulation (C–H NF) using ionic gelation method. C–H NF exhibited elite physico-biochemical properties, influencing physiological and biochemical dynamics in Tomato. These elite properties include homogenous-sized nanoparticles (314.4 nm), lower PDI (0.218), viscosity (1.43 Cps), higher zeta potential (11.2 mV), nanoparticle concentration/ml (3.53 × 10^8^), conductivity (0.046 mS/cm), encapsulation efficiency (53%), loading capacity (24%) and yield (32.17%). FTIR spectroscopy revealed histidine interaction with C–H NF, while SEM and TEM exposed its porous structure. Application of C–H NF to Tomato seedling and potted plants through seed treatment and foliar spray positively impacts growth parameters, antioxidant-defense enzyme activities, reactive oxygen species (ROS) content, and chlorophyll and nitrogen content. We claim that the histidine-functionalized chitosan nanoformulation enhances physico-biochemical properties, highlighting its potential to elevate biochemical and physiological processes of Tomato plant.

## Introduction

Biodegradable nanomaterials are attracting more interest due to their elite physiological characteristics that enhance transportation efficiency, reduce environmental impact and toxicity, promote human health, and support ecological balance in natural environments^1–3^. Among the various nanomaterials, chitosan biopolymer based nanomaterials are highly researched in the agriculture sector by encapsulation of various active ingredients (AIs) viz. pesticides, plant hormones, and fertilizers^4–7^. Encapsulation of AIs helps to amplify the use efficiency of AIs for plant growth and protection. Encapsulations have been done to achieve slow, control, and target deliveries which are the main characteristic of chitosan nanomaterials for their application in plant^8^. Further, the immense physico-biochemical activity of the chitosan biopolymer lies in its functional group (C-6 –OH and –NH_2_). These functional groups provide a broad spectrum platform for enhancing chitosan functionality by attaching AIs^9,10^. Modification in the functional group significantly alters the size, shape, polydispersity, size distribution, and surface charge of chitosan nanoparticles^11^. Further, modification changes of these physico-biochemical properties considerably affect the biological activity of nanoparticles in plants. In addition, to enhance the bioactivity of chitosan nanoformulations, many AIs have been explored^8^. Various amino acids have been used as AIs to change the properties of chitosan viz. arginine, alanine, lysine, proline, histidine, and glutamic acid to raise chitosan-based formulations for use in medical, pharmaceutical, and agriculture^12^. Among the amino acids, histidine is the most functionally diverse amino acid which has an imperative role in plant metabolism as a structural and functional component^13,14^. Histidine is involved in vital biological processes in plants, including protein synthesis, enzymatic activities, and pH regulation, serving as a proton transfer mediator in various proteins, and participating in numerous molecular interactions. Its biocompatibility with plants is facilitated by interactions with receptors on the postsynaptic membrane, leading to the opening of amino acid-gated channels^13,14^. Chitosan-based formulations incorporated histidine has been documented through diverse methodologies including hydration, crosslinking, fluorimetric, and phase inversion method^12^. These formulations have demonstrated applicability in various fields, such as metal ion absorption as evidenced by studies conducted^15–18^. Furthermore, the versatility of these formulations extends to drug delivery applications^19^. Additionally, research has explored their utility in gene delivery^20–22^. Moreover, the chitosan-based formulations have shown promise in cancer therapy^23^, and in the realm of wound healing^24^. However, the method of ionic gelation in which sodium tripolyphosphate (TPP) has been considered to be the method where the payload potential of chitosan nanoparticles increases many folds^8^. This study focuses on the preparation of novel and highly active histidine functionalized chitosan nanoformulations designed to enhance its functionality through the modulation of physico-biochemical properties of nanoparticles. To the best of our knowledge, there haven’t been established formulations of this kind of combination as bioactive material ever before in the agriculture field. This study provides a comprehensive elucidation of the synthetic methodology and evaluates the influence of varied histidine concentrations on the physicochemical properties of chitosan-histidine nanoformulation (C–H NF). Furthermore, the synthesized nanoformulation underwent evaluation in Tomato test crop, employing both seed treatment and foliar application, to quantify its biochemical and physiological impacts on the plants.

## Results

### Synthesis of C–H NF

In this study, C–H NF synthesis employed the ionic gelation method by crosslinking of 0.5% chitosan and 0.5% TPP. Various histidine concentrations were explored for interaction with the chitosan + TPP complex. Functional groups of histidine interacted with the complex, resulting in a compact nanoscale chitosan-histidine nanoformulation (C–H NF). Among concentrations (0.5, 1, 1.5, 2, 2.5, and 3%; w/v), 2% histidine exhibited compatibility based on the DLS study. After freeze-drying, approx 386 mg of C–H NF dry powder was obtained per reaction (210 mL).

### Physicochemical characterization of C–H NF

#### DLS, EE, LC, in vitro release profile, yield, viscosity, and NTA study

**Figure 1:**
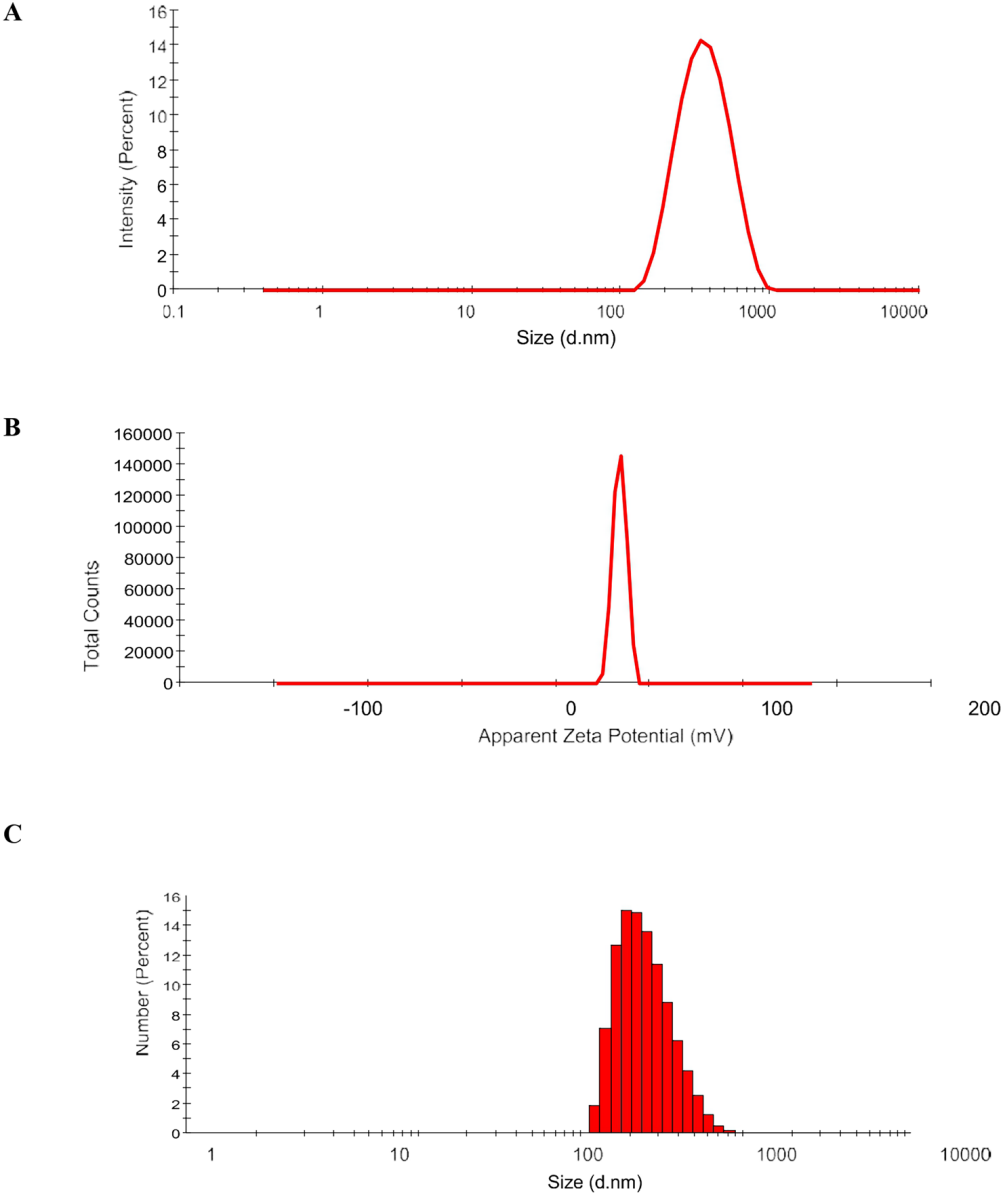
DLS analysis of C-H NF for (**A**) size (**B**) zeta-potential and (**C**) size distribution by number.

**Figure 2:**
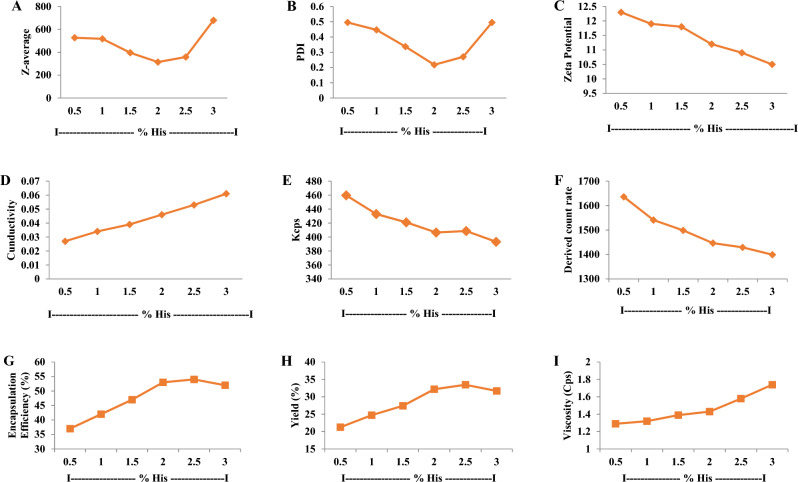
Effect of different concentrations of histidine (0.5–3%) on (**A**) z-average (**B**) PDI (**C**) zeta potential (**D**) conductivity (**E**) Kcps (**F**) Derived count rate (**G**) encapsulation efficiency (%) (**H**) yield (%) and (**I**) viscosity of chitosan-histidine nanoformulation (C–H NF).

**Figure 3:**
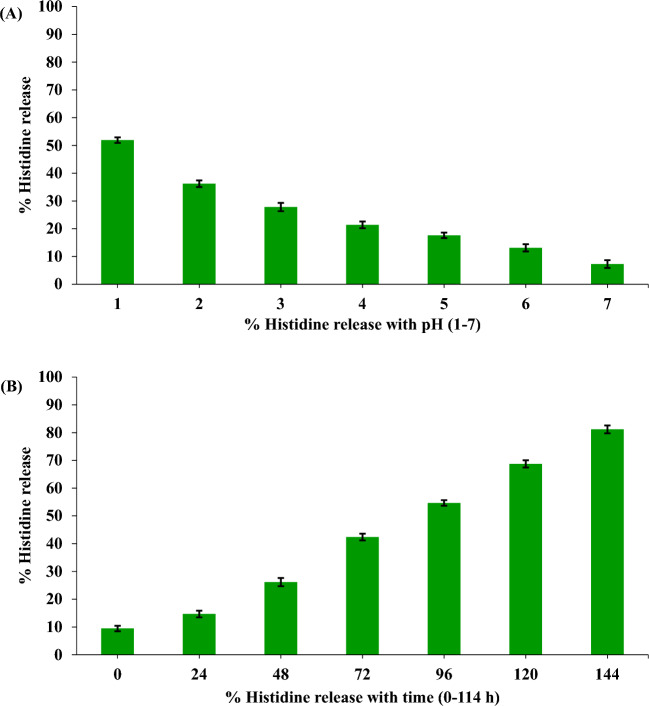
In vitro release of Histidine from positively charged C–H NF at different pH (**A**) and time (**B**). Each value is mean of triplicates and each replicate consisted of 3 samples.

**Figure 4:**
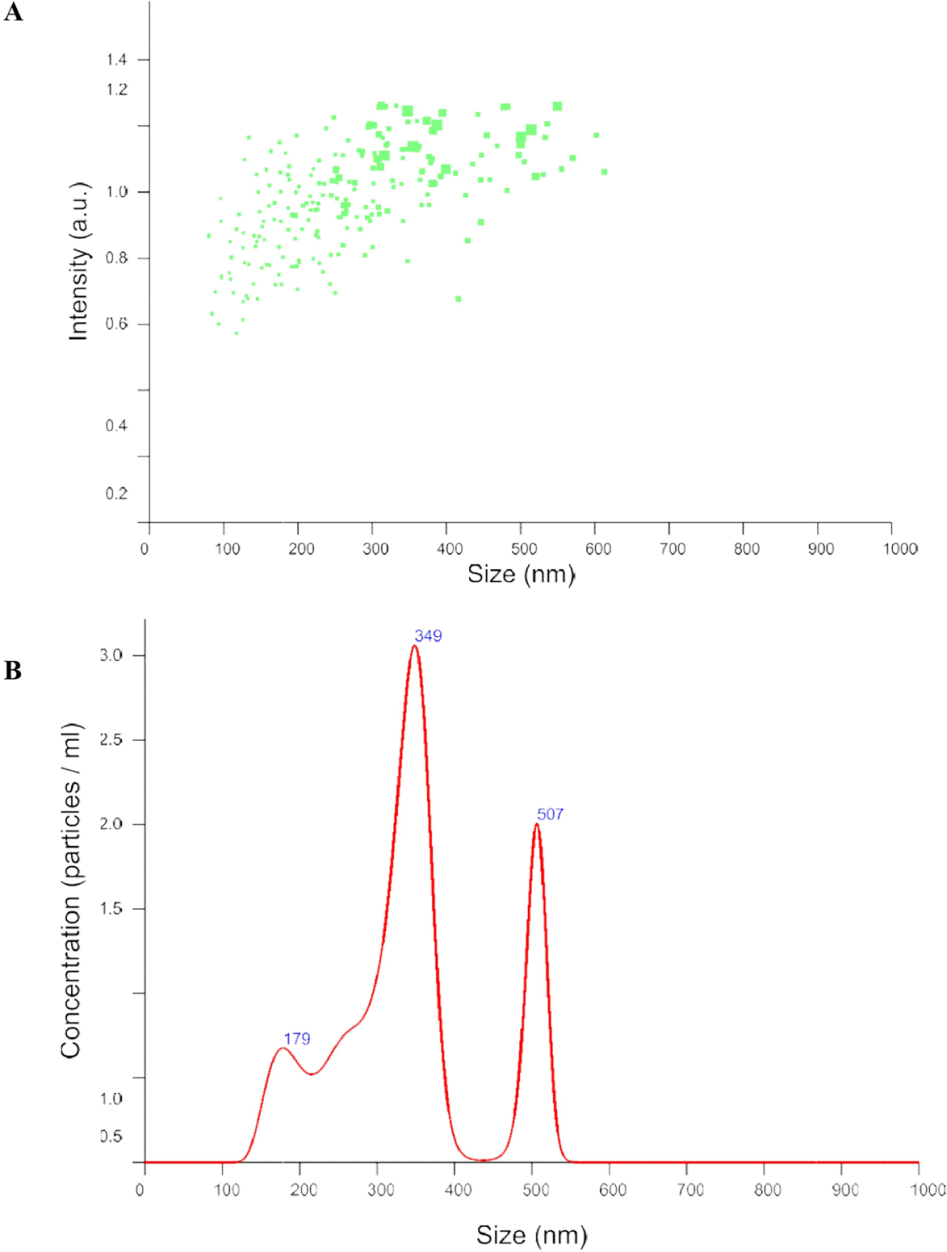
NTA analysis of C–H NF (**A**) size (**B**) concentration of particle/mL.

C-H NF was synthesized with varying histidine concentrations (0.5–3%; w/v). At 2% histidine, the nanoparticles exhibited a size of 314.4 nm, PDI of 0.218, zeta potential of 11.2 mV, conductivity of 0.046 mS/cm, viscosity of 1.43 Cps, kcps of 406.5, DCR of 1446.4, encapsulation efficiencies of 53%, Loading capacity (LC) of 24% and yield percentage of 32.17% (Figs. 1, 2A–I). The release profile analysis revealed a notable decline in the rate of histidine release from C-H NF as the pH increased from 1 to 7, with values decreasing from 51.9 to 7.26%. Particularly, the release rate was observed to be considerably faster within the pH range of 1–3 (Fig. 3A). Additionally, over time, there was a gradual rise in the release of NH_2_+ , reaching 81.16% after 144 h (Fig. 3B). Further characterization via NTA study revealed 3.53 × 10^8^ C-H NF nanoparticles per mL with a mean size of 341.6 ± 0.00 nm at pH 7 in an aqueous solution (Fig. 4).

#### FTIR, SEM and TEM of C–H NF

**Figure 5:**
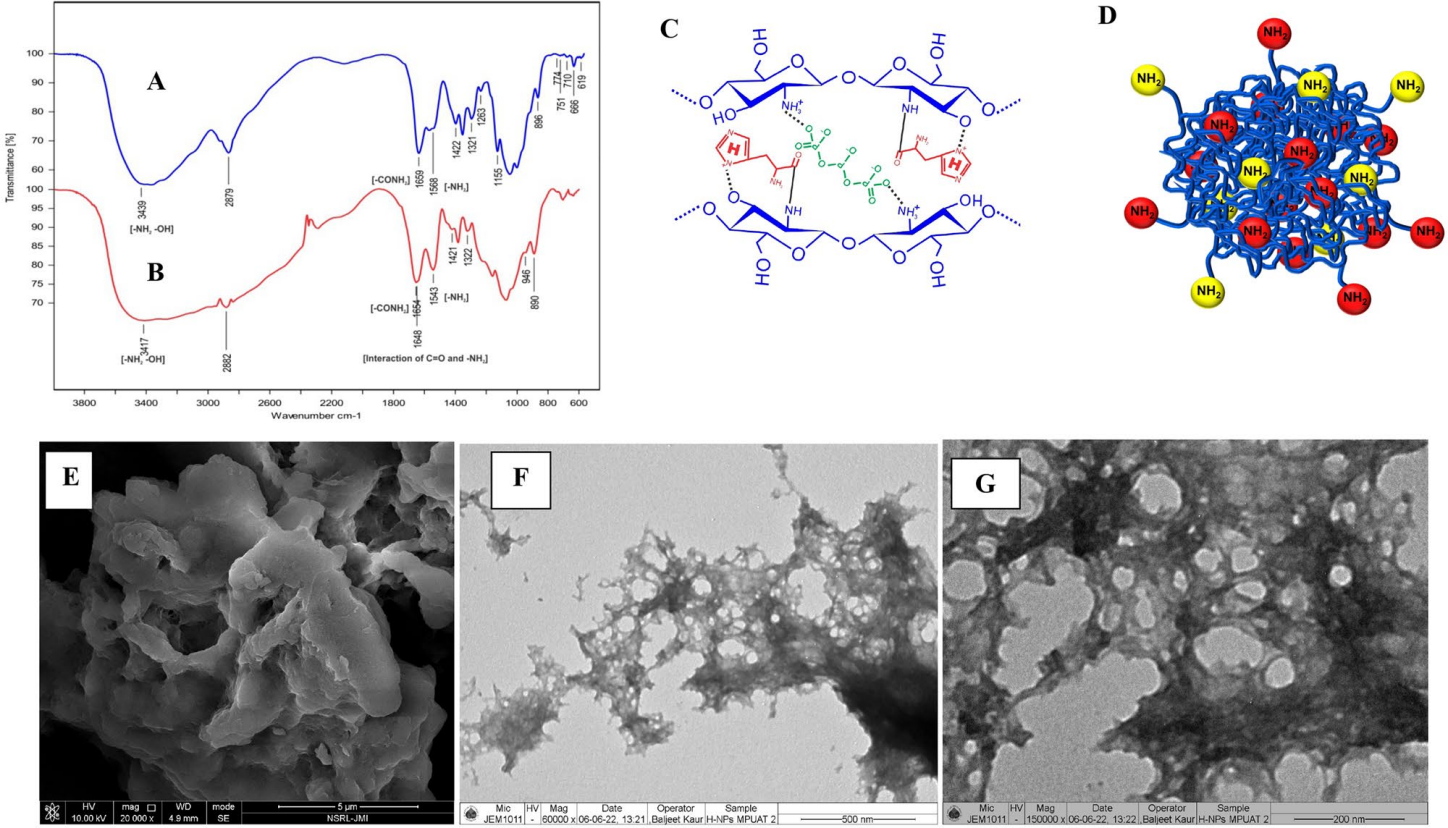
FTIR spectra of (**A**) bulk chitosan (**B**) C–H NF (**C**), (**D**) hypothetical model of C–H NF showing interactions of chitosan, TPP and histidine and SEM and TEM study of C–H NF, SEM at (**E**) 20 kx, TEM at (**F**) 60 kx (**G**) 150 kx Source: generated through CorelDRAW Graphics Suite 2024, Version 12; URL: Graphic design software with CorelDRAW Graphics Suite (https://www.coreldraw.com/en/product/coreldraw/)).

Nanoformulations underwent FTIR analysis for functional group assessment. The main peak at 3439 cm^-1^ in bulk chitosan shifted to 3417 cm^-1^ in C-H NF, indicating alterations in –NH_2_ and –OH groups (Fig. 5A and B). Peaks shifted 1654 (–CONH_2_), 1543 (–NH_2_), and 890 cm^-1^ (anhydrous glycosidic bond) relative to bulk chitosan, revealing chitosan–TPP interaction. A peak at 1648 cm^-1^ in C–H NF corresponding to C=O of histidine interacted with –NH_2_ of chitosan. Peaks at 2879 cm^-1^ intensified in C–H NF, suggesting the amidation of chitosan. The possibility of histidine’s imidazole ring interacting through the –CN to –OH group of chitosan was indicated by the peak shift from 3439 to 3417 cm^-1^. Further, SEM and TEM studies confirmed the surface morphology and internal structure of C-H NF. SEM (EVO MA10, Carl Zeiss Promenade, Jena, Germany) revealed a spherical nanomaterial with a porous structure (Fig. 5E). However, HR-TEM (HR-TEM, JEOL, JEM 2100F, USA) showed mild pores throughout the structure (Fig. 5F and G).

#### *In-vitro* seedling growth

The study investigated the impact of varied C-H NF concentrations (0.001, 0.003, 0.006, 0.012, and 0.015%, w/v) on seedling growth. After a 10-day germination period, results showed a notable positive effect on Tomato seedlings, with a significantly higher germination percentage (95.9–99.8%). C–H NF concentrations ranging from 0.006 to 0.015% showed significant improvement in shoot length (5.42–25.58%), root length (14.94–60.92%), and SVI (25.90–42.52%) compared to histidine alone (Table 1).

**Table 1:** Effects of C-H NF on tomato seedling growth in in-vitro.

Treatments (%)	Seed germination (%)	Shoot length (cm)	Root length (cm)	Seedling vigor index (SVI)
Control (Water)	84.6 ± 0.49^g^	8.7 ± 0.14^g^	5.7 ± 0.23^h^	1221.6 ± 19.6^i^
BCH (0.01)	91.8 ± 0.57^f^	10.1 ± 0.05^f^	7.3 ± 0.16^ g^	1601.5 ± 23.3^h^
Histidine (0.01)	93.9 ± 0.60^de^	12.9 ± 0.77^d^	8.7 ± 0.07^ef^	2033.0 ± 49.4^f^
C-H NF				
0.001	91.8 ± 0.49^f^	11.4 ± 0.49^e^	8.0 ± 0.03f.	1794.8 ± 31.9^g^
0.003	93.8 ± 0.57^d^	14.9 ± 0.61^b^	9.9 ± 0.06^e^	2367.4 ± 30.8^d^
0.006	97.9 ± 0.45^b^	16.2 ± 0.72^a^	10.0 ± 0.07^d^	2580.7 ± 42.7^c^
0.009	99.8 ± 0.66^a^	15.4 ± 0.49^ab^	13.8 ± 0.49^a^	2895.1 ± 67.5^a^
0.012	99.3 ± 0.61^a^	13.6 ± 0.60^c^	13.9 ± 0.49^a^	2748.1 ± 22.0^b^
0.015	95.9 ± 0.60^c^	13.6 ± 0.45^c^	12.9 ± 0.45^b^	2556.6 ± 36.6^c^
0.018	94.9 ± 0.60^cd^	11.8 ± 0.49^e^	12.3 ± 0.45^bc^	2295.2 ± 41.9^e^

Various growth parameters were recorded at 15 days of tomato seedling. Each value is mean of triplicates and each replicate consisted of 10 seedlings. Error bars represent mean ± SE (standard error) with the same letter statistically insignificant (*p* = 0.05) as determined by Tukey–Kramer HSD. Water (control) and BCH (bulk chitosan, 0.01% dissolved in 0.1% acetic acid).

### Plant growth parameters

**Figure 6:**
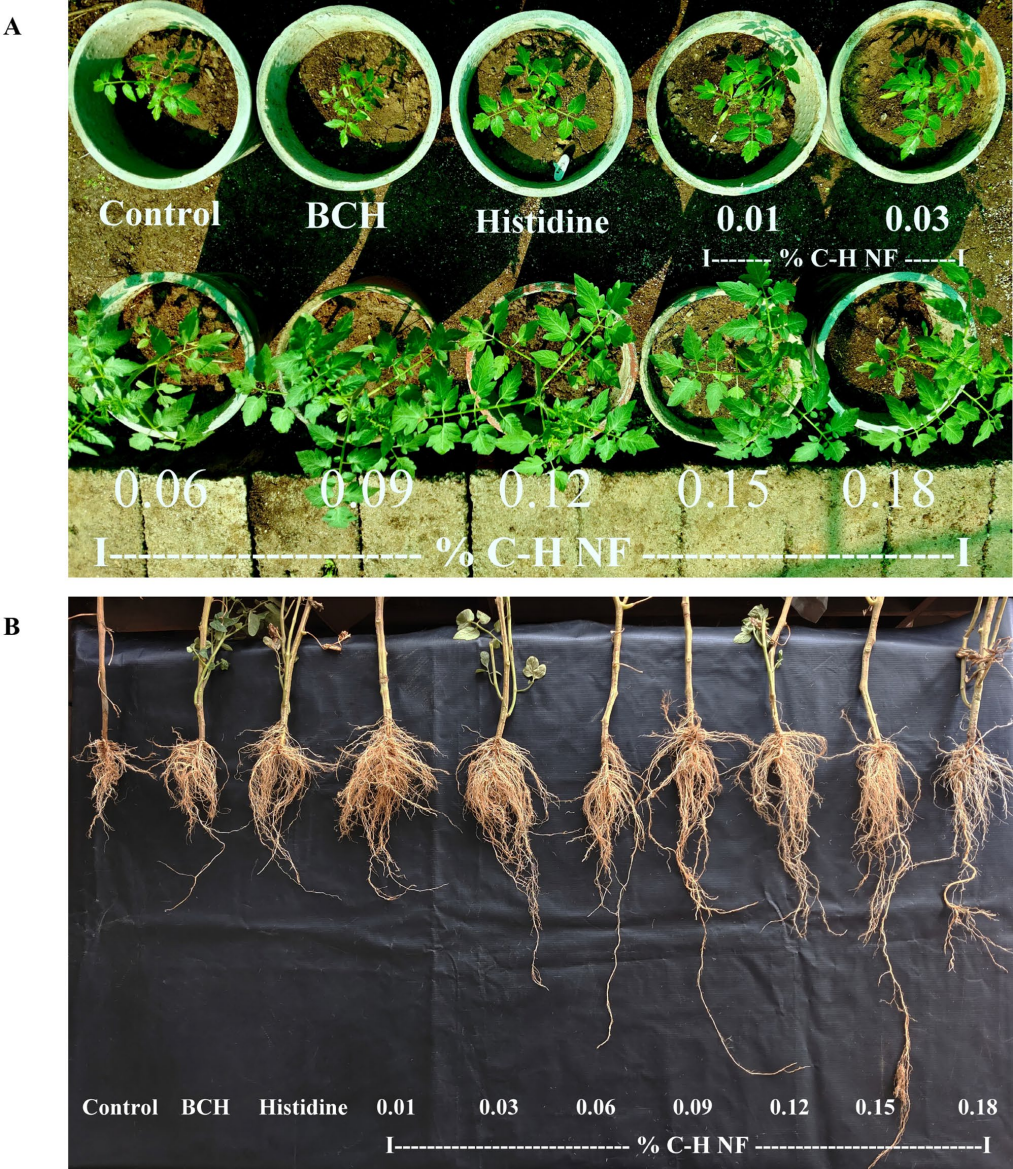
Effect of C-H NF on (**A**) plant growth (**B**) root growth of Tomato in pot experiment.

Growth parameters, including plant height, branch and leaves count per plant, stem diameter, leaf area, and root length were assessed at physiological maturity. In the pot experiment, 0.03–0.15% C-H NF concentration significantly increased plant height (10.31–53.25%) compared to histidine (Table 2; Fig. 6A). However, the number of branches/plant (19.19–50.51%), and leaves/plant (19.65–43.46%) with all concentrations exceeded histidine (Table 2). Additionally, C–H NF (0.01–0.18%) showed effectiveness in stem diameter (5.41–27.03%), leaf area (1.20–10.91%), and root length (3.46–79.16%) compared to histidine, (Table 2; Fig. 6B). Notably, 0.03–0.15% C–H NF concentration demonstrated effectiveness across all growth parameters in the pot experiment.

**Table 2:** Effect of C-H NF on growth parameters of tomato in pot plant.

Treatments (%)	Plant height (cm)	Number of branch/plant	Number of leaves/plant	Stem diameter (mm)	Leaf area (cm^2^)	Root length (cm)
Control (Water)	43.9 ± 2.01^i^	6.9 ± 0.49^g^	35.9 ± 0.92^f^	6.0 ± 0.17^c^	293.2 ± 1.42^ g^	14.1 ± 0.87^i^
BCH (0.01)	51.0 ± 1.55^h^	8.9 ± 0.60^f^	47.6 ± 1.61^e^	6.4 ± 0.21^c^	300.4 ± 1.09f.	27.6 ± 0.67^h^
Histidine (0.01)	59.2 ± 0.66^f^	9.9 ± 0.16^e^	51.1 ± 0.74^d^	7.4 ± 0.21^b^	309.2 ± 1.47^e^	29.3 ± 0.57^g^
C–H NF						
0.01	58.9 ± 0.75^fg^	11.8 ± 0.77^d^	62.3 ± 0.71^bc^	7.8 ± 0.24^b^	315.9 ± 2.05^d^	30.2 ± 2.15^f^
0.03	65.3 ± 1.28^e^	12.4 ± 0.60^c^	61.1 ± 2.38^bc^	8.1 ± 0.03^b^	312.8 ± 1.71^de^	33.0 ± 1.23^e^
0.06	69.9 ± 2.30^d^	11.9 ± 0.49^d^	61.8 ± 2.48^bc^	8.5 ± 0.08^ab^	316.8 ± 0.74^d^	42.1 ± 1.13^c^
0.09	70.9 ± 0.63^d^	14.4 ± 0.61^a^	63.9 ± 2.40^b^	7.8 ± 0.37^b^	325.7 ± 1.01^bc^	48.6 ± 1.08^ab^
0.12	73.7 ± 1.20^c^	13.6 ± 0.61^ab^	64.4 ± 2.31^ab^	8.4 ± 0.21^ab^	320.3 ± 1.09^cd^	32.6 ± 1.08^e^
0.15	91.0 ± 1.57^a^	14.9 ± 0.60^a^	73.3 ± 1.12^a^	9.4 ± 0.10^a^	343.2 ± 3.22^a^	52.7 ± 1.25^a^
0.18	85.4 ± 1.63^ab^	12.9 ± 0.16^c^	65.8 ± 1.41^ab^	8.1 ± 0.14^b^	329.8 ± 0.52^b^	37.9 ± 0.76^d^

Growth parameters were recorded at physiological maturity of tomato pot plants under shade net condition. Each value is mean of triplicates and each replicate consisted of 3 plants. Error bars represent mean ± SE (standard error) with the same letter statistically insignificant (*p* = 0.05) as determined by Tukey–Kramer HSD. Water (control), BCH (bulk chitosan, 0.01% dissolved in 0.1% acetic acid) and histidine (0.01%, w/v).

### Antioxidants and defense enzymes, ROS, chlorophyll, and nitrogen content

Following foliar application on pot plants, C-H NF (0.01–0.18% w/v) was examined for antioxidant, defense enzyme activities, ROS, and chlorophyll and N content. In the pot experiment, SOD activity (15.79–68.42%) with all C-H NF concentration was significantly higher than histidine, control, and BCH (Table 3). Likewise, POD (36.96–86.96%), CAT (8.33–50%), and PPO (6.67–40%) activities with all C–H NF concentrations were found significantly higher than histidine (Table 3). Conversely, H_2_O_2_, O_2_^–1^ content in C-H NF (0.01 to 0.18%) was markedly lower than control, BCH, and histidine (Table 3). Total chlorophyll content (14.29–57.14%) increased notably with all concentrations compared to histidine (2.17 mg/g), control, and BCH (Table 3). N content (1.62–2.02%) was significantly higher with C-H NF (0.01–0.18%) compared to control (1.40%) (Table 3). Overall, all C–H NF concentrations exhibited effectiveness in pot plant experiments for antioxidant, defense enzyme activities, ROS, and chlorophyll content.

**Table 3:** Effect of C–H NF on antioxidants and defense enzymes, ROS, chlorophyll and nitrogen content of tomato in pot plant.

Treatments (%)	SOD (µmol/min/g)	POD (µmol/min/g)	CAT (mmol/min/g)	PPO (µmol/min/g)	H_2_O_2_ (µmol/g)	O_2_^-^ (µmol/g)	Chlorophyll (mg/g)	Nitrogen (%)
Control (Water)	0.07 ± 0.00^i^	0.27 ± 0.01^ h^	0.4 ± 0.07^j^	1.2 ± 0.01^i^	197.8 ± 5.0a	67.0 ± 1.01a	1.5 ± 0.05^i^	1.40 ± 0.02^ h^
BCH (0.01)	0.10 ± 0.00^ h^	0.32 ± 0.02^ g^	1.1 ± 0.05^i^	1.4 ± 0.03^ h^	190.3 ± 8.0b	62.9 ± 3.05b	1.9 ± 0.07^ h^	1.70 ± 0.04^ef^
Histidine (0.01)	0.19 ± 0.01^ g^	0.46 ± 0.03f.	2.4 ± 0.06^ h^	1.5 ± 0.02^ g^	182.7 ± 4.0c	58.7 ± 2.04c	2.1 ± 0.06^ g^	2.30 ± 0.05^a^
C-H NF								
0.01	0.22 ± 0.01f.	0.63 ± 0.01^e^	2.6 ± 0.05^ g^	1.6 ± 0.03f.	176.8 ± 6.0d	54.2 ± 4.03d	2.4 ± 0.06f.	1.74 ± 0.03^e^
0.03	0.24 ± 0.00^e^	0.63 ± 0.04^e^	2.7 ± 0.08f.	1.7 ± 0.05^d^	171.6 ± 2.7e	48.5 ± 2.75e	2.4 ± 0.09^e^	1.84 ± 0.02^c^
0.06	0.28 ± 0.00^c^	0.66 ± 0.02^d^	3.2 ± 0.09^d^	1.6 ± 0.03f.	163.9 ± 4.0f.	43.4 ± 1.53f.	2.8 ± 0.05^d^	2.02 ± 0.06^b^
0.09	0.30 ± 0.01^b^	0.63 ± 0.03^e^	3.0 ± 0.07^e^	2.0 ± 0.04^b^	162.8 ± 7.0f.	36.7 ± 3.09 g	2.9 ± 0.09^c^	1.74 ± 0.04^e^
0.12	0.26 ± 0.01^d^	0.85 ± 0.02^b^	3.3 ± 0.09^c^	2.1 ± 0.03^a^	154.9 ± 2.4 g	30.1 ± 2.43 h	3.1 ± 0.07^b^	1.79 ± 0.05^ cd^
0.15	0.32 ± 0.00^a^	0.86 ± 0.04^a^	3.6 ± 0.09^a^	2.0 ± 0.02^bc^	145.3 ± 4.0 h	26.2 ± 3.37i	3.3 ± 0.04^a^	1.62 ± 0.03^ g^
0.18	0.28 ± 0.0^0c^	0.77 ± 0.01^c^	3.4 ± 0.08^b^	1.7 ± 0.01^e^	165.2 ± 5.0f.	36.1 ± 1.64 g	2.4 ± 0.09f.	1.62 ± 0.03^ g^

Effect of C–H NF on antioxidants and defense enzymes, ROS, chlorophyll and nitrogen content were measured at 3rd day of second foliar application in tomato pot plants under shade net condition. Each value is mean of triplicates and each replicate consisted of 3 plants. Error bars represent mean ± SE (standard error) with the same letter statistically insignificant (*p* = 0.05) as determined by Tukey–Kramer HSD. Water (control), BCH (bulk chitosan, 0.01% dissolved in 0.1% acetic acid).

## Discussion

The ionic gelation method is employed for the synthesis of C–H NF, wherein TPP serves as a crosslinker^26^. Through this approach, nanomaterials can be effortlessly tailored to achieve desired size ranges, PDI, and zeta-potential^8,42^. The positively charged functional groups (–NH_3_^+^) of chitosan (0.5%) in an acidic medium interacted with the negatively charged –PO_4_^-^ (phosphate) group of 0.5% TPP (ionic cross-linker), leading to the formation of crosslinked chitosan + TPP complex. The procedure is primarily characterized by ionic cross-linking. In this approach, various concentrations of histidine (0.5, 1, 1.5, 2, 2.5, and 3%; w/v) are used to interact with the chitosan + TPP complex. During the crosslinking process, some –NH_3_^+^ groups remain unbound and accessible for subsequent interaction with histidine. The functional groups of histidine (–C=O and –C–N) further interacted with the chitosan + TPP complex, resulting in the formation of a condensed nanoscale C-H NF. Based on the FTIR study, an amidation reaction, occurring between amine groups (–NH) of chitosan and carboxyl group (–C=O) of histidine produced the chitosan-histidine as represented in Fig. 5A and B^19^. Among the various concentrations of histidine, 2% histidine was chosen based on the findings of the DLS study (Fig. 2). After freeze drying, approximately 386 mg C–H NF dry powder was obtained from each reaction. Unlike chitosan, the synthesized nanoformulation was completely dissolved in distilled water.

**Figure 7:**
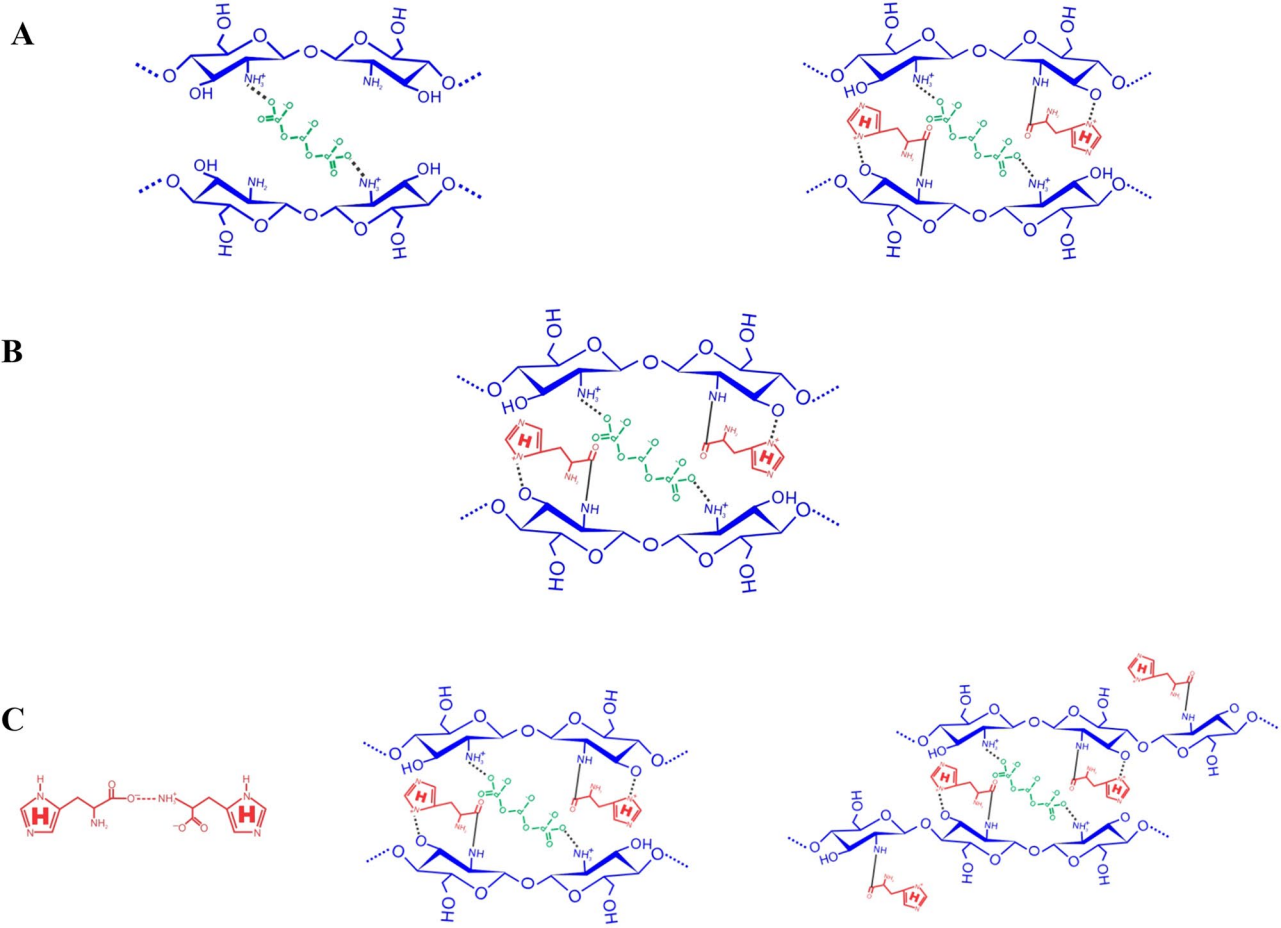
Hypothetical model representing interaction of CS, TPP and His. (Source: generated through CorelDRAW Graphics Suite 2024, Version 12; URL: Graphic design software with CorelDRAW Graphics Suite (https://www.coreldraw.com/en/product/coreldraw/)).

The optimization of the final histidine concentration for the synthesis of chitosan-histidine nanoformulation was systematically conducted, taking into account multiple parameters, including z-average (nm), PDI, zeta potential (mV), conductivity (mS/cm), kcps, and DCR, encapsulation efficiency (EE), loading capacity (LC) as well as in vitro release profile. The nanoformulations synthesized at concentrations ranging from 0.5 to 1.5% exhibited z-average values for diameter, ranging from 527.1 to 396.3 nm and PDI ranging from 0.495 to 0.337 (Fig. 2A and B). Moreover, in the context of EE and LC, it is crucial to assess the degree of interaction between the chitosan-TPP complex and histidine within the C–H NF. To assess the EE and LC, a ninhydrin test was performed on the supernatant obtained during the purification step of synthesis to estimate the free amino acid content. The encapsulation efficiency ranged from 37 to 47%, while the yield ranged from 21.26 to 27.42% within this concentration range (0.5–1.5%) (Fig. 2G and H). Furthermore, in the concentration range of 0.5–1.5%, the formulation's viscosity and conductivity demonstrated an increase (Fig. 2D and I), attributed to the saturation of all charged functional groups associated with chitosan and histidine^19^. It was noted that an increased propensity for chitosan-TPP interactions was discerned in contrast to the chitosan-TPP-histidine complex (Fig. 7A). This is attributed to the insufficient amount of histidine available for interaction with chitosan-TPP during ionic gelation. Consequently, this deficiency led to diminished encapsulation efficiency and yield. Furthermore, within the histidine concentrations range of 2.5–3%, an escalation in the z-average was noted, rising significantly from 358.7 to 678.6 nm. Concurrently, the PDI value, viscosity, and conductivity also exhibited an increasing trend (Fig. 2A, B, D and I). It is postulated that, under these conditions, a chitosan-TPP-histidine complex, along with a more intricate chitosan-TPP-histidine structure, was formed (Fig. 7C). Moreover, at these concentrations, interactions between histidine molecules (histidine-histidine) were observed due to the elevated histidine concentration (Fig. 7C). The escalation in PDI was attributed to excess histidine facilitating intermolecular interactions between histidine molecules^43^. However, the encapsulation efficiency and yield percentage of the formulation decreased at histidine concentrations surpassing 2.5% (Fig. 2G and H). This reduction is attributed to the interaction of his-his, coupled with multiphase interactions of chitosan-TPP-histidine due to the surplus availability of histidine. Some studies have documented that an excess of histidine in aqueous solutions leads to the formation of hydrogen bonding and dipeptide bonds between histidine–histidine^43,44^. Furthermore, the concentration range of 0.5–3% (Fig. 2C), illustrates a slight reduction in the zeta potential with an increase in concentration. The observed phenomenon suggests that the zeta potential is responsive to variations in histidine concentration, indicative of its dependence on the presence of influenced by the availability of free functional groups in nanoformulations. The increased concentration of histidine led to the occupancy of the –NH_2_ group of chitosan, despite the presence of its functional group in the form of the protonated –NH_2_ group of the imidazole ring.

Among the various histidine concentrations investigated, a 2% concentration was employed to synthesize C–H NF. The resulting C–H NF exhibited z-average of 314.4 nm, a PDI of 0.218, a zeta potential of 11.2 mV, conductivity of 0.046 mS/cm, viscosity of 1.43 Cps, kilo-counts per second (kcps) of 406.5, derived count rate (DCR) of 1446.4, encapsulation efficiencies of 53%, and yield percentage of 32.17% (Fig. 2A-I). Lower PDI and viscosity may enhance the interaction between nanoparticles and plant cells, facilitating nutrient absorption. Further, the conductivity of the solution was influenced by the concentration of histidine in the solution. In addition, the primary influences on diminishing the size of the chitosan particles were marked maximum in the solution’s viscosity and conductivity. Size and zeta potential are critical characteristics influencing the interaction of nanomaterials with plant cells, impacting physico-biochemical responses in plants^45^. Positive zeta potential in C–H NF is noteworthy, as it contributes to the stability of nanoformulations in aqueous solutions and enhances their interaction with negatively charged plant cell surfaces^30^. The significantly lower PDI value at 2% histidine concentration indicates that the nanoformulation predominantly consisted of the chitosan-TPP-histidine complex (Fig. 7B). The positive zeta potential of the prepared nanoformulation along with the low PDI indicates its colloidal stability for use in agriculture applications. A lower PDI signifies a homogeneous distribution of particle sizes, preventing the agglomeration of smaller particles into larger ones. The uniformity in nanoparticle sizes is crucial for facilitating facile entry into plant cells through cellular passages and surface openings^46^. Furthermore, maintaining a lower PDI (0.180) not only ensures stability but also augments the overall bioactivity^47,48^. Maintaining this reduced PDI is crucial for stability and increased bioactivity, as underscored in studies^49,50^. C–H NF displayed slow release of histidine (Fig. 3A and B). With a decrease in pH from 3 to 1, the histidine release escalated from 27.80% to 51.90%, suggesting accelerated release at acidic pH due to chitosan protonation (see Fig. 3A)^55^. It is postulated that the uniform-sized nanoparticles contributed significantly to the regulated histidine release from the nanoformulations^49^. The C–H NF, formulated with 2% histidine, underwent further characterization through a Nanoparticle Tracking Analysis (NTA). This investigation revealed a substantial concentration of nanoparticles, quantified at 3.53 × 10^8^ particles per mL, with an average size of 341.6 nm at pH 7 in an aqueous environment (Fig. 4A and B). The efficacy of nanoformulations in cellular systems is contingent upon achieving a higher particle concentration per unit volume of the formulation^8^. In our research, the notable concentration per mL of C–H NF (3.53 × 10^8^) signifies efficacy in enhancing higher biological activities.

The FTIR spectra facilitated the identification of intermolecular connections among the functional groups present in chitosan, histidine, and C-H NF (Fig. 5A and B). Specifically, the FTIR spectrum of chitosan (Fig. 5A) exhibited distinctive absorption bands at characteristic wavenumbers, including 3439 cm^−1^ (attributed to –NH_2_ and –OH stretching), 1659 (indicative of –CONH_2_ bending), 1568 (corresponding to –NH_2_ bending), and 896 cm^−1^ (associated with the anhydrous glycosidic bond). The major characteristic peak at 3417 cm^−1^ observed in the FTIR spectrum of C-H NF is found to correlate with the –NH_2_ and –OH groups inherent in the bulk chitosan present in C-H NF (Fig. 5A and B). Furthermore, a notable shift in peak position is evident, with the peaks moving to 1654 (reflecting –CONH_2_), 1543 (indicative of –NH_2_), and 890 cm^−1^ (associated with the anhydrous glycosidic bond) as compared to bulk chitosan. This observed shift in peak positions provides valuable insight into the interaction dynamics between chitosan and TPP, underscoring the structural alteration in the formation of C–H NF^30,46^. The FTIR analysis of C-H NF revealed a distinctive peak at 1648 cm^-1^, corresponding to the C=O group of histidine, indicating an interaction with the –NH_2_ group of chitosan^19,51^. Additionally, in chitosan, the peaks at 2879 cm^−1^ intensified in C-H NF, shifting to 2882 cm^−1^, suggesting amidation of chitosan. Furthermore, a plausible interaction involving the imidazole ring of histidine and the –OH group of chitosan was inferred from the shift in the peak from 3439 to 3417 cm^−1^. Based on these findings, a hypothetical structure model of C-H NF was proposed, illustrating the various interactions (Fig. 5C and D). Subsequently, SEM (EVO MA10, Carl Zeiss Promenade, Jena, Germany) and TEM (HR-TEM, JEOL, JEM 2100F, USA) studies confirmed the surface morphology and internal structure of the nanoformulations, respectively. SEM study showed a spherical-shaped nanomaterial with a porous structure (Fig. 5E). However, the TEM study revealed that it has mild pores over the structure (Fig. 5F and G). The high porosity of the external and internal surface of nanomaterial gives a higher surface area which is responsible for more interaction with the plant surface and surface receptors^8,51^. In previous studies, similar external and internal architecture was confirmed in chitosan and TPP-based chitosan nanoformulations^8,42^.

The current investigation focused on the impact of varying concentrations of C–H NF (0.001, 0.003, 0.006, 0.012, and 0.015%, w/v) on seedling growth with observation recorded after a 10-day germination period. The results indicate a noteworthy influence on Tomato seedlings, with a significantly higher germination % (95.9–99.8%). Additionally, the C–H NF exhibited significant improvements in shoot length (5.42–25.58%), root length (14.94–60.92%), and SVI (25.90–42.52%) in 0.006–0.015% concentrations of C-H NF over the histidine (Table 1).

In the current research, the supplementations of C-H NF resulted in a noteworthy enhancement of growth characteristics in Tomato plants during a potted experimental regimen. This effect was observed to be superior compared to untreated control, bulk chitosan, and histidine (Table 2). Considering the growth promoting there was a massive difference between the C-H NF and its bulk form. The efficacy of the nanoformulation surpassed that of the bulk form, even when applied at same concentrations (0.01). The results unveiled an augmentation in growth attributed to the application of C–H NF across all the parameters. These parameters include plant height, number of leaves per plant, number of branches per plant, leaf area, stem diameter, and root length. The growth-enhancing effect was observed across a concentration range of 0.01–0.18%. However, at a 0.15% concentration of the nanoformulation, maximum values were observed for all growth parameters (Table 2; Fig. 6). C–H NF has been engineered to enable the gradual release of histidine to plants via seed treatment and foliar application, with the aim of enhancing plant growth. The beneficial effects of C–H NF on Tomato plants may be attributed to its inherent physicochemical properties, such as uniform-sized nanoparticles, reduced polydispersity index (PDI), elevated zeta potential, and enhanced nanoparticle concentration/mL. Notably, the biochemical and physiological responses of Tomato plants to C–H NF could result from a synergistic interplay between histidine and chitosan. Specifically, C–H NF contains a total of four nitrogen atoms, with three derived from histidine and one from chitosan. Consequently, the nanoformulation has the potential to act as an organic nitrogen source for Tomato plants, thereby potentially stimulating improved growth. The activity of SOD ranges from 0.22 to 0.32 µmol/min/g, significantly surpassing the levels in the control (0.07 µmol/min/g), BCH (0.10 µmol/min/g) and histidine (0.19 µmol/min/g) across all concentration of C–H NF (Table 3). Similarly, the activities of POD (0.63–0.86 µmol/min/g), CAT (2.6–3.6 mmol/min/g), and PPO (1.6–2.1 µmol/min/g) were higher in all concentrations of C-H NF compared to histidine, BCH and control (Table 3). Further, foliar application of chitosan nanoformulations extensively mitigated ROS levels and maintained cellular homeostasis by fostering the activities of antioxidant and defense enzymes. However, the content of H_2_O_2_ (176.8–145.3 µmol/g) and O_2_^–1^ (54.2–26.2 µmol/g) in all concentrations of C–H NF (0.01–0.18%) was prominently lower as compared with control, BCH and histidine (Table 3). The higher activity of chitosan nanoformulations in treated plants effectively counteracted the level of ROS (H_2_O_2_ and O_2_^−^) in the leaves^49,52,53^. Previous studies have revealed the role of increased antioxidant enzyme activity in reducing ROS level in the plants^46,50^. Our findings align with this, as we observed that higher activity of SOD, POD, CAT, and PPO significantly controlled the concentration of H_2_O_2_ and O_2_^-^ in potted Tomato plants. Furthermore, the total chlorophyll content (2.41–3.30 mg/g) of pot plants in all the concentrations of C–H NF (0.01–0.18) exhibited noteworthy enhancement compared to the control (1.56 mg/g), BCH (1.94 mg/g), and histidine (2.17 mg/g) (Table 3). Additionally, the N content (1.62–2.02%) was significantly higher in all concentrations of C–H NF (0.01–0.18%) compared to the control (1.40%) (Table 3). The increased levels of chlorophyll and nitrogen are important for the higher rate of photosynthesis, contributing to enhanced plant growth p and yield^42^.

Numerous reports have documented the development of chitosan nanoformulations employing the ionic gelation method with emphasis on the utilization of TPP^8,30,42,48,49,54,55^. These nanoformulations have been used to enhance plant immunity against diseases and growth for getting an adequate yield. However, limited literature is available where chitosan histidine nanoparticles have been developed without TPP for metal ion absorption^15–18^, drug delivery^19^, gene delivery^20–22^, cancer therapy^23^, and wound healing^24^. To the best of current knowledge, chitosan histidine nanoformulations have not undergone evaluation for their impact on physiological and biochemical responses in plants. In the current investigation, a chitosan nanoformulation functionalized with histidine was explored, revealing notable changes in the physico-biochemical properties of C-H NF. Previous studies on chitosan nanomaterials have reported various ranges of PDI ranging from 0.3 to 0.5^8^. In present investigation, the PDI was significantly lower as compared to the previously reported chitosan nanomaterials, supporting its higher physiological and biochemical impact on Tomato plants, likely attributed to its homogenous interaction with plant cells. Previous investigations have demonstrated elevated zeta potential in chitosan nanoformulation, attributed to the presence of freely available positively charged –NH_2_ group in chitosan^8,49,54,55^. In the current formulation (C–H NF), histidine has been incorporated, introducing its functional group with a positive charge. The addition of this functional group has not significantly changed the zeta potential, resulting in a balanced zeta potential. However, higher encapsulation efficiency and lower PDI have been observed. The concentration of nanomaterials in aqueous solutions is crucial for maximizing their effectiveness in plants. It is very important for its higher activity in plants and the determination of concentration of nanoparticles has been reported previously on chitosan nanomaterials^46^. In our study, a considerably higher nanoparticle concentration/mL was recorded through NTA analysis. The higher concentration of nanoparticle/mL is presumed to have contributed to the higher bioactivity of nanoformulation in Tomato plants. Moreover, the presence of an FTIR peak at 1648 cm^−1^ in C–H NF confirmed the interaction between –C=O of histidine and –NH_2_ group of chitosan through amide linkage^19^. Furthermore, the –CN group of histidine (Imidazole ring) may have interacted with the –OH group of chitosan, facilitated by the protonation of the –NH group of the imidazole ring in an acidic medium^19,56^. Furthermore, the observed biological activity in Tomato plants can be attributed to two facts. The physico-biochemical properties of chitosan-histidine nanoformulations, characterized by a lower z-average and PDI value, along with a positive zeta potential, are considered influential. Furthermore, higher conductivity, kcps, DCR, and nanoparticle concentration/mL contribute to enhanced growth and development. Moreover, the potential biological activity of histidine in the plants cannot be discounted, as its presence entails three N atoms that may serve as an N source of the plant and function as an organic N source. Previous studies have also suggested the role of histidine in plant metabolism as both a structural and functional component^13,14^.

## Conclusion

The current research asserts that the anticipated positive impact of C–H NF on Tomato plants can be attributed to its inherent physico-chemical properties, including homogenous-sized nanoparticles, lower PDI, higher zeta potential, and higher nanoparticle concentration/mL. More specifically, the biochemical and physiological responses of Tomatoes to C–H NF may occur through a synergetic effect involving histidine and chitosan. To elaborate further, C–H NF contained a total four nitrogen atom, three from histidine and one from chitosan. Consequently, the nanoformulation could act as a potential organic nitrogen source for Tomatoes, potentially contributing to enhanced growth. Notably, at a dosage of 0.009%, C–H NF prompted positive effects on seedling growth in the Petri plate. Moreover, in potted plants, the foliar spray of C–H NF (0.15%) proved effective across all growth parameters. It maintained antioxidant-defense enzyme activities, balanced ROS content, and considerably enhanced chlorophyll and nitrogen content. According to the findings, the current study claims that the application of C–H NF can be implemented in field experiments for translation and validation of technology. In the future, the novel histidine functionalized chitosan nanoformulation holds promise across various fields. Advanced synthesis methods may refine its properties, enhancing its efficacy in biomedical applications such as targeted drug delivery and tissue engineering. In agriculture, expect tailored formulations to revolutionize crop protection, improving plant resilience to pests and diseases while promoting sustainable farming practices. These innovations could significantly boost plant growth, yield, and quality, leading to more robust crops and higher agricultural productivity. Moreover, the nanoformulation’s versatility could extend to environmental applications, such as textile dye removal or heavy metal ions removal, addressing pressing challenges in pollution mitigation.

## Materials and methods

### Materials

Chitosan of low molecular weight (50,000–190,000 Da, 80% degree of deacetylation), sodium tripolyphosphate (TPP), and histidine were procured from Sigma-Aldrich, St. Louis, MO, USA. Other chemicals were sourced from HiMedia and SRL, Mumbai, India. Deionized (DI) water was used in the study. Seeds of Tomato cultivar ‘NBH-2453’ were used from Nobel Seeds Pvt. Ltd., India.

### Synthesis of chitosan–histidine nanoformulations

To synthesize C-H NF, the ionic gelation method was explored^25,26^. Accordingly, 0.5% chitosan was dissolved in glacial acetic acid (1% v/v) to get a pH of 3.84. Thereafter, NaOH solution was then used to adjust the processed chitosan (0.5%) to maintain pH 5.10 followed by cross-linking with 0.5% TPP. The cross-linking was conducted using a pediatric set (Romsons, Agra, India) under a magnetic stirrer (200 rpm; Remi Laboratory Instruments, Mumbai, India). Before completing the cross-linking, the chitosan-TPP complex further interacted with different concentrations of histidine solutions (0.5, 1, 1.5, 2, 2.5, and 3%; w/v). The final concentration of histidine was optimized based on the DLS study. The formulation was further centrifuged (10,000 rpm for 10 min), and precipitated semisolid material was resuspended in DI water and sonicated (Q 500 Sonicator Qsonica, USA) for 4 min with 5-s pulse on/off, 40% amplitude. This process was repeated three times. The obtained semi-solid formulation was lyophilized (FreeZone 6, Labconco, USA), and stored at room temperature.

### Characterization of chitosan–histidine nanoformulations

Chitosan-histidine nanoformulations were investigated for comprehensive characterization utilizing dynamic light scattering (DLS) on a Zetasizer instrument (ZS90, Malvern, U.K.). Measurements were done at 25 °C, employing a scattering angle of 90 °C. Key parameters including Z-averages, size distribution, PDI, zeta potential, conductivity, kcps, and DCR were determined through triplicate measurements, ensuring robust and reliable data acquisition. Different concentrations of histidine solutions, ranging from 0.5 to 3% (w/v), were used for investigation. The optimization of the final histidine concentration was selected based on a thorough analysis using DLS studies. Additionally, mean size and particle concentration (number of particles/mL) were validated through NTA (Nano Sight NS300, Malvern Panalytical, UK). The interaction among functional groups in C-H NF was assessed via Fourier transform infrared spectroscopy (FTIR; Alpha, Bruker, Germany). Lyophilized nanoformulations with potassium bromide (KBr) were triturated (in 1:99 ratios) and spectra were collected in transmittance mode at 25 °C in the 3800–600 cm^−1^ region. SEM (scanning electron microscopy), and TEM (transmission electron microscope) analyses were conducted to examine the surface morphology and internal structure. For SEM (EVO MA10, Carl Zeiss Promenade, Jena, Germany), samples were dried using critical point drying (CPD, Emitech K850) and coated with gold–palladium via a sputter coating^21^. For TEM (HR-TEM, JEOL, JEM 2100F, USA), nanoformulations were diluted with deionized water, sonicated (2 cycles) with a probe sonicator, negatively stained with 2% phosphotungstic acid (PTA) and dried at room temperature (25 ± 2 °C) before observation^28^.

### Encapsulation efficiency (EE), loading capacity (LC), in vitro release profile, yield, and viscosity of C-H NF

The EE and LC of histidine in the nanoformulations were assessed using the Ninhydrin test via the spectrophotometer method^29,30^. Experiments were performed to explore the impact of pH and time duration on the release dynamics of histidine from C-H NF. Freeze-dried C–H NF samples were dispersed in 50 mL of ultrapure water adjusted to pH levels ranging from 1 to 7. Subsequently, centrifugation at 10,000×*g* for 10 min was conducted. Similarly, C–H NF dispersed in 50 mL of deionized water at pH 4.5 were subjected to incubation for durations of 0, 24, 48, 72, 96, 120, and 144 h, followed by centrifugation at 10,000×*g* for 10 min. The resultant supernatants from both sets of experiments were subjected to analysis for histidine content utilizing a spectrophotometric technique at 570 nm employing the ninhydrin test^29,30^. The yield of C–H NF was calculated based on the various concentrations of histidine (0.5, 1, 1.5, 2, 2.5, and 3%) used during the synthesis of the nanoformulation. EE was calculated by following equation^30^.

$${\text{EE}} = \frac{{\text{Free amount of histidine in suparnatant}}}{{\text{Total amount of histidine}}} \times 100$$

$${\text{LC}} = \frac{{\text{Free amount of histidine in suparnatant}}}{{{\text{Weight of C}} - {\text{H NF}}}} \times 100$$

The viscosity was described by the Ostwald viscometer method^31^. Viscosities were calculated using the formula:

$$\frac{{\eta }_{s}}{{\eta }_{w}}= \frac{{\rho }_{s}}{{\rho }_{w}} \frac{{t}_{s}}{{t}_{w}}$$

### *In-vitro* seedling growth

The seedling growth of Tomato variety ‘NBH 2453’ was investigated in a Petri plate using standard methods with minor adjustments^32^. Briefly, the seeds underwent a 1-h treatment with various concentrations of C–H NF (0.001, 0.003, 0.006, 0.009, 0.012, 0.015, and 0.018% w/v), in addition to control (water), bulk-chitosan (0.01%; w/v) and histidine (0.01%; w/v) all dissolved in DI water. In this experiment, 15 days post-sowing, parameters such as seed germination percentage, shoot–root length, and seedling vigour index (SVI) were assessed. The seedling vigour index (SVI) was calculated using the formula described^33^.

$${\text{Seedling vigour index }}\left( {{\text{SVI}}} \right) \, = {\text{ Germination }}\% \, \times \, \left( {{\text{root length }} + {\text{ shoot length}}} \right)$$

### Plant growth, antioxidants and defense enzymes, ROS, and chlorophyll content

A pot experiment was carried out from July to October 2022–23 at the research farm of Rajasthan College of Agriculture, Maharana Pratap University of Agriculture and Technology, Udaipur, India (24.58° latitude, 73.70° longitude). The experiment followed a randomized block design (RBD) with three replications. Different concentrations of C-H NF were assessed through foliar spray in a pot experiment conducted under net house conditions. Tomato seeds were sown in earthen pots filled with standard potting soil (clay type soil; pH 8.2, EC. 0.56 dSm^−1^) sourced from the field. The synthesized nanoformulation (0.01, 0.03, 0.06, 0.09, 0.12, 0.15, and 0.18% w/v), bulk chitosan (0.01%), histidine (0.01%), and control (water) underwent two foliar applications: the first 10 days after transplanting and the second just before flowering stage. Plant growth parameters viz. plant height, number of leaves per plant, number of branches per plant, leaf area, stem diameter, and root length were recorded at the time of physiological maturity. Portable leaf area meter (Model: Li-3000) was used to measure leaf area from tagged Tomato plant and was expressed as cm^2^. Antioxidant and defense enzymes like superoxide dismutase (SOD) (EC 1.15.1.1), peroxidase (POD) (EC 1.11.1.7), catalase (CAT) (EC 1.11.1.6) and polyphenol peroxidase (PPO) (EC 1.10.3.1) were estimated 3 days after 2nd foliar application of various treatments^34–36^. The H_2_O_2_ and O_2_^−^^37,38^, chlorophyll^39^, and nitrogen content^40^ were measured after 3 days of 2nd foliar application.

### Statistical analysis

Statistical analyses were performed with JMP software version 12 employing the Kramer HSD test at a significance level of *p* = 0.05^41^. All experiments were executed in triplicate, with each replication comprising a minimum of ten samples (for seedling experiments) and three samples (for pot experiments) selected randomly from the plant population.

## Resources

The study was in accordance with relevant institutional, national, and international guidelines and legislation.

## Data Availability

All relevant data generated or analysed are included in the manuscript and the supporting materials.
